# The Rice CHD3/Mi-2 Chromatin Remodeling Factor Rolled Fine Striped Promotes Flowering Independent of Photoperiod

**DOI:** 10.3390/ijms22031303

**Published:** 2021-01-28

**Authors:** Hyeryung Yoon, Yejin Shim, Soo-Cheul Yoo, Kiyoon Kang, Nam-Chon Paek

**Affiliations:** 1 Department of Agriculture, Forestry and Bioresources, Plant Genomics and Breeding Institute, Research Institute of Agriculture and Life Sciences, Seoul National University, Seoul 08826, Korea; hyeryungyoon@snu.ac.kr (H.Y.); yejin5648@snu.ac.kr (Y.S.); 2 Department of Plant Life and Environmental Science, Hankyong National University, Anseong 17579, Korea; scyoo@hknu.ac.kr; 3 Division of Life Sciences, Incheon National University, Incheon 22012, Korea

**Keywords:** *Rolled Fine Striped* (*RFS*), flowering time, rice (*Oryza sativa*), ATP-dependent chromatin remodeling factor, epigenetics, histone methylation

## Abstract

Genetic studies have revealed that chromatin modifications affect flowering time, but the underlying mechanisms by which chromatin remodeling factors alter flowering remain largely unknown in rice (*Oryza sativa*). Here, we show that Rolled Fine Striped (RFS), a chromodomain helicase DNA-binding 3 (CHD3)/Mi-2 subfamily ATP-dependent chromatin remodeling factor, promotes flowering in rice. Diurnal expression of *RFS* peaked at night under short-day (SD) conditions and at dawn under long-day (LD) conditions. The *rfs-1* and *rfs-2* mutants (derived from different genetic backgrounds) displayed a late-flowering phenotype under SD and LD conditions. Reverse transcription-quantitative PCR analysis revealed that among the flowering time-related genes, the expression of the major floral repressor *Grain number and heading date 7* (*Ghd7*) was mainly upregulated in *rfs* mutants, resulting in downregulation of its downstream floral inducers, including *Early heading date 1* (*Ehd1*), *Heading date 3a* (*Hd3a*), and *Rice FLOWERING LOCUS T 1* (*RFT1*). The *rfs* mutation had pleiotropic negative effects on rice grain yield and yield components, such as plant height and fertility. Taking these observations together, we propose that *RFS* participates in multiple aspects of rice development, including the promotion of flowering independent of photoperiod.

## 1. Introduction

In rice (*Oryza sativa*), flowering time is a critical yield-related trait that is important for seasonal and regional adaptation worldwide. The transition from the vegetative to the reproductive phase is promoted by two florigen genes in rice, *Heading date 3a* (*Hd3a*) and *Rice FLOWERING LOCUS T 1* (*RFT1*), which are first expressed in the leaves [1,2]. Hd3a and RFT1 act as the major mobile signals, moving to the shoot apical meristem and triggering the transition from vegetative to reproductive growth. *Heading date 1* (*Hd1*) acts as a floral inducer or repressor depending on the length of the photoperiod [3]. *Hd1* upregulates the expression of *Hd3a* and *RFT1* under short-day (SD) conditions and downregulates them under long-day (LD) conditions. *Early heading date 1* (*Ehd1*) encodes a B-type response regulator that upregulates the expression of *Hd3a* and *RFT1* [4].

A number of regulators upstream of *Ehd1* have been identified. For instance, *Grain number and heading date 7* (*Ghd7*), encoding a CCT domain protein, downregulates *Ehd1* expression in LDs [5]. *Early heading date 2* (*Ehd2*)/*Oryza sativa Indeterminate 1* (*OsID1*)/*Rice Indeterminate 1* (*RID1*) promotes flowering by upregulating *Ehd1* expression in both SD and LD conditions [6,7,8]. *Early heading date 3* (*Ehd3*), encoding a PHD finger protein, downregulates *Ghd7* expression in LDs and upregulates *Ehd1* expression in LD and SD conditions [9]. The rice gene *CONSTANS-LIKE 4* (*OsCOL4*) acts as a down-regulator of *Ehd1* independent of the photoperiod [10].

Histone methylation is mediated by the histone methyltransferase (HMT) catalytic activity of SET DOMAIN GROUP (SDG) proteins, and the resulting alterations of chromatin structure affect the expression of flowering genes in rice [11]. For instance, methylation of histone H3 lysine 4 (H3K4) or lysine 36 (H3K36) on the chromatin of the flowering-related genes *Hd3a*, *RFT1*, and *Ehd1* is required for promotion of flowering, but methylation of histone H3 lysine 27 (H3K27) represses the expression of flowering genes. The knockdown of *SET DOMAIN GROUP 708* (*SDG708*) by RNA interference decreases mono-, di-, and trimethylation of histone H3K36 (H3K36me1/me2/me3) on the *Hd3a*, *RFT1*, and *Ehd1* loci, leading to late flowering [12]. SDG701 trimethylates H3K4 (H3K4me3) in *Hd3a* and *RFT1* chromatin to activate the floral transition [13]. A rice Trithorax group protein, OsTrx1, recruits the WD40 protein OsWDR5a and SDG723/OsTrx1/OsSET33 Interaction Protein 1 (SIP1) to deposit H3K4me3 on the *Ehd1* locus [14,15,16]. SDG724 mediates the deposition of H3K36me2/me3 to upregulate the expression of *OsMADS50* and *RFT1* [17]. Mutation of *SDG725* results in late flowering via a reduction of H3K36me2/me3 marks on several flowering genes including *Ehd2*, *Ehd3*, *OsMADS50*, *Hd3a*, and *RFT1* [18,19]. Polycomb repressive complex 2 (PRC2) downregulates the expression of two genes encoding flowering repressors, rice *Late Flowering* (*OsLF*) and *LEAFY COTYLEDON 2* and *FUSCA 3-LIKE 1* (*OsLFL1*), via trimethylation of histone H3 lysine 27 (H3K27me3) [20,21,22].

In addition to epigenetic regulation of SDG proteins, ATP-dependent chromatin remodeling factors are also involved in altering chromatin structure to modulate flowering time. In *Arabidopsis thaliana*, the putative sucrose non-fermenting (SNF2)-like ATPase subunit PICKLE (PKL) plays roles in the floral transition by regulating the expression of the floral meristem identity gene *LEAFY* (*LFY*) and gibberellic acid (GA)-regulated genes [23,24]. PKL physically interacts with CONSTANS (CO) to facilitate binding to *FLOWERING LOCUS T* (*FT*) chromatin. PKL mediates deposition of H3K4me3 on the *FT* locus by associating with ARABIDOPSIS HOMOLOG OF TRITHORAX 1 (ATX1) to antagonize Polycomb group (PcG)-mediated repression of *FT* [25,26]. CHROMATIN REMODELING 4 (CHR4) interacts with transcription factors involved in floral meristem identity to regulate the expression of key floral regulators [27].

In rice, Rolled Fine Striped (RFS), which encodes a chromodomain helicase DNA-binding 3 (CHD3)/Mi-2 chromatin-remodeling factor, is involved in various aspects of rice development such as crown root development, seedling development, and leaf morphogenesis [28,29,30]. Genome-wide analysis revealed that the *rfs* mutant shows a reduction of H3K4me3 and H3K27me3 [31]. These results demonstrate that RFS can modulate both active and repressive epigenetic marks on histones. Our previous report showed that RFS controls reactive oxygen species (ROS) homeostasis by modulating H3K4me3 levels on ROS-related genes [32]. 

Here, we found that two *rfs* mutants (*rfs-1* and *rfs-2*) derived from different genetic backgrounds exhibited late flowering independent of the photoperiod, implying that *RFS* is involved in flowering-time regulation in rice. Although numerous genes that control rice flowering have been reported, few epigenetic regulator that modulate flowering has not been identified in rice. To reveal the regulatory function of *RFS* in rice flowering, we analyzed the expression and histone methylation levels of flowering-time genes in *rfs-2* mutant. Reverse transcription-quantitative PCR (RT-qPCR) showed that *RFS* promotes flowering by downregulating *Ghd7* in LDs and upregulating *Ehd1* in SD and LD conditions. Our results suggest a new molecular function of *RFS* as a floral regulator in rice.

## 2. Results

### 2.1. The rfs Mutants Exhibit a Late Flowering Phenotype Independent of the Photoperiod

**Figure 1:**
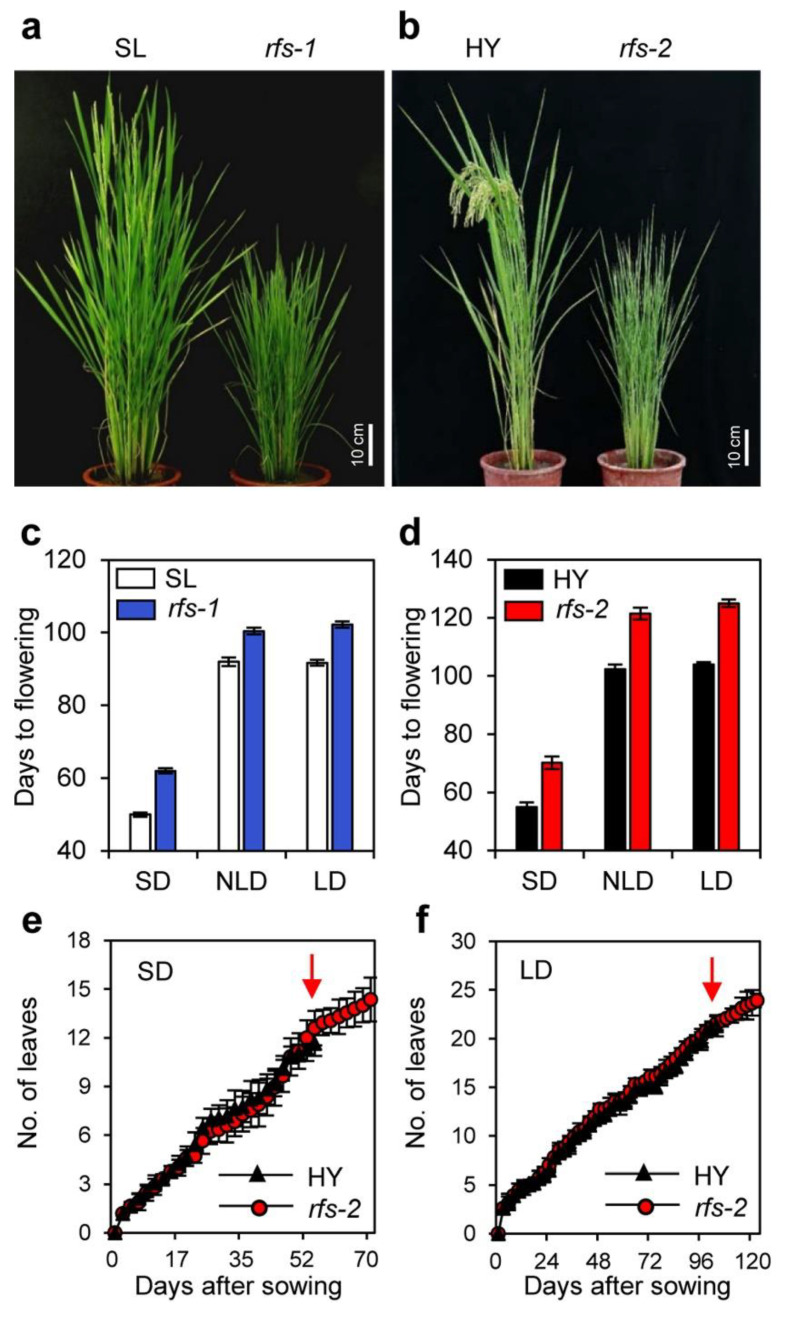
Mutation of *RFS* results in delayed rice flowering independent of the photoperiod. (**a**,**b**) Flowering phenotypes of the *rfs-1* (**a**) and *rfs-2* (**b**) mutants were compared with their parental *japonica* cultivars ‘Seolak’ (SL), and ‘Hwayoung’ (HY), respectively. Rice plants were grown in the paddy field under natural long-day (NLD) (~14 h light/day) conditions until the parental lines flowered. White scale bars = 10 cm. (**c**,**d**) Days to flowering of the *rfs-1* (**c**) and *rfs-2* (**d**) mutants and their parental lines (SL and HY) were determined in short-day (SD) (10 h light/day), NLD, and long-day (LD) (14.5 h light/day) conditions. (**e**,**f**) Comparison of leaf emergence rates between HY and *rfs-2* plants under SD (**e**) and LD (**f**) conditions during plant development. The leaf emergence rate was scored according to the method of Itoh et al. (1998). Red arrows indicate the average flowering date of HY. Means and standard deviations were obtained from 20 plants (**c**,**d**) and 10 plants (**e**,**f**) of each genotype.

To elucidate the involvement of *RFS* in flowering-time regulation in rice, we investigated the flowering time of two *rfs* mutants [32] under different photoperiod conditions. The *rfs-1* mutant (which was isolated from a gamma-ray mutagenesis) flowered around 8 d later than its parental *japonica* cultivar ‘Seolak’ (SL), which flowered at 92 days after sowing (DAS) under natural long-day (NLD) conditions in the field (37° N latitude, Suwon, Korea). In the growth chambers, the *rfs-1* mutant flowered around 8 d and 11 d later than SL under SD (10 h light/14 h dark), and LD (14.5-h light/9.5-h dark) conditions, respectively (Figure 1a,c). 

To further confirm whether mutation of *RFS* delays rice flowering, we observed the flowering time of the *rfs-2* knockout mutant, which harbors a T-DNA fragment in the 8th intron of *RFS* [32]. Similar to the *rfs-1* mutant, the flowering time of the *rfs-2* mutant was delayed by 19 d compared with its parental *japonica* cultivar ‘Hwayoung’ (HY), which flowered at 102 DAS. In addition, the *rfs-2* mutant flowered around 15 d and 21 d later than HY under SD, and LD conditions, respectively (Figure 1b,d). These observations suggest that *RFS* is involved in the promotion of flowering, independent of photoperiod.

To test whether growth retardation causes the late flowering in *rfs* mutants, we measured the number of emerged leaves in HY and *rfs-2* plants under SD and LD conditions [33]. The leaf emergence rates of the *rfs-2* mutant were indistinguishable from those of HY under both conditions (Figure 1e,f). Therefore, the late flowering of *rfs* mutants was mainly due to a delayed floral transition, not to growth retardation or prolonged plastochron.

### 2.2. Expression Pattern of RFS

**Figure 2:**
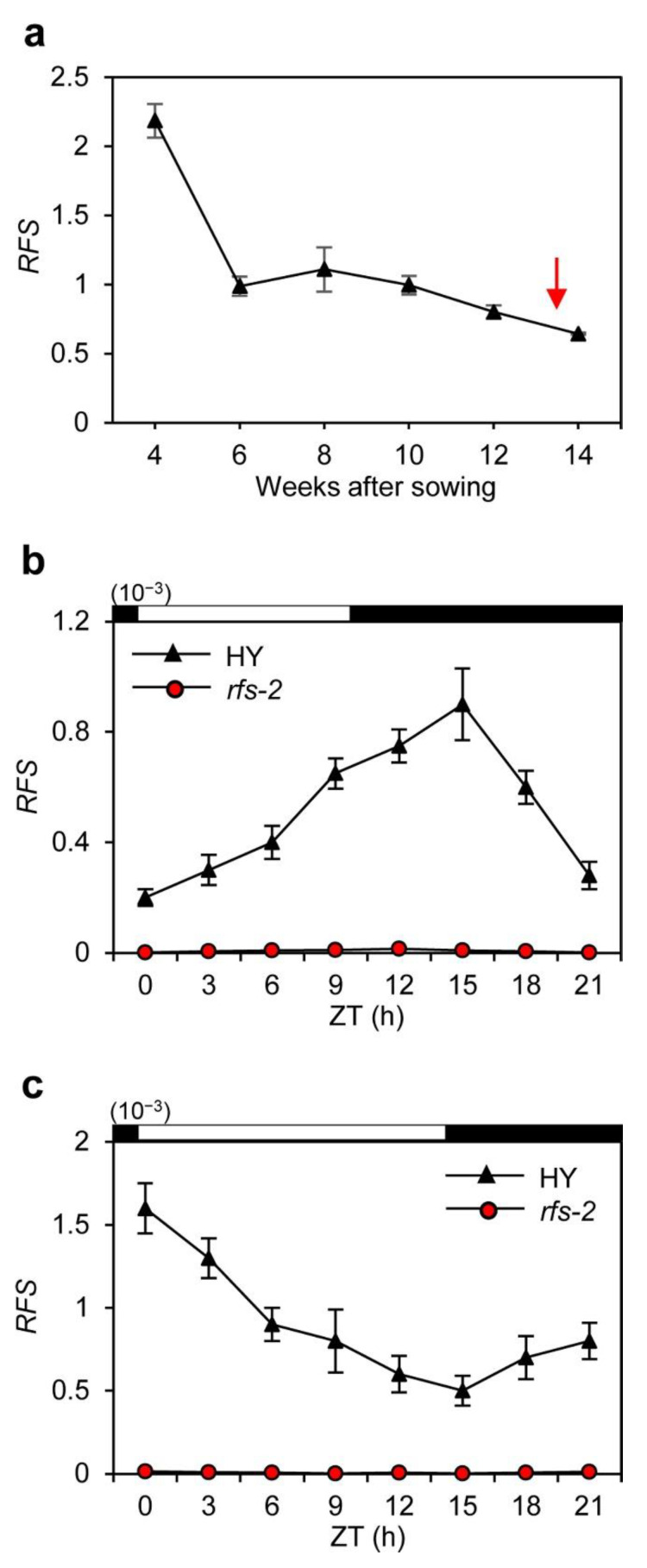
Expression profile of *RFS*. (**a**) Expression levels of *RFS* during plant development in HY under LD conditions. Total RNA was isolated from the leaves harvested every 2 weeks from 4 weeks after sowing (WAS) to 14 WAS at ZT1. The red arrow indicates the average flowering date of HY. (**b**,**c**) Diurnal change of *RFS* expression in HY and *rfs-2* plants under SD (**b**) and LD (**c**) conditions. Total RNA was extracted from HY and *rfs-2* leaf blades harvested every 3 h during a 24-h period from plants at 25 days after sowing (DAS) in SDs and 80 DAS in LDs. The open and filled bars at the top of graphs represent light and dark periods, respectively. Transcript levels of *RFS* were determined by RT-qPCR and normalized to that of *OsUBQ5* (*LOC_Os01g22490*). Means and standard deviations were obtained from three biological replicates. Experiments were repeated three times with similar results. ZT, zeitgeber time (hours after dawn).

The *RFS* transcript levels were measured every 2 weeks from 4 to 14 weeks after sowing (WAS) at zeitgeber time (ZT) 1 by RT-qPCR analysis. *RFS* was constitutively expressed during plant growth, implying that RFS has roles throughout plant development (Figure 2a). We also analyzed the diurnal expression of *RFS* under SD and LD conditions. The leaf blades of HY and *rfs-2* plants were harvested every 3 h during a 24-h period at 25 DAS under SD conditions and at 80 DAS under LD conditions; these timepoints were approximately 30 days before flowering in HY. *RFS* transcripts were not detectable in *rfs-2* leaf blades, but in HY, *RFS* showed diurnal expression patterns with a peak at night under SD conditions and at dawn under LD conditions (Figure 2b,c). 

### 2.3. Expression Patterns of Hd3a and RFT1 in the rfs Mutants 

**Figure 3:**
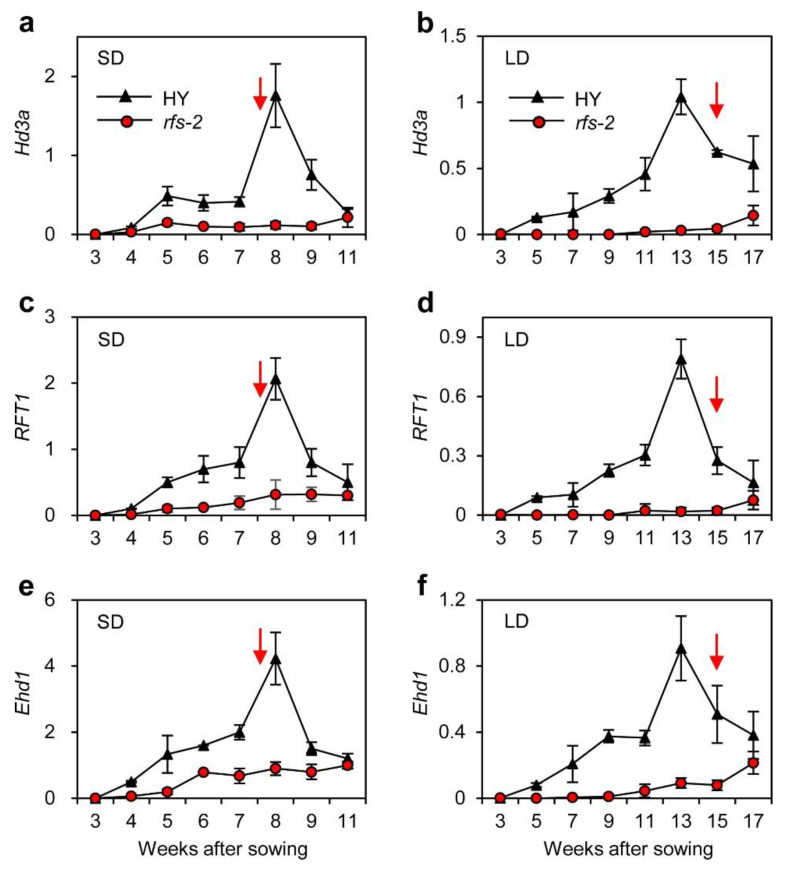
Altered expression of flowering genes in the *rfs-2* mutant during plant development. Total RNA was isolated from HY and *rfs-2* leaf blades collected 1 h after dawn (ZT1) in SD (**a**,**c**,**e**) and LD (**b**,**d**,**f**) conditions. Transcript levels of *Hd3a* (**a**,**b**), *RFT1* (**c**,**d**), and *Ehd1* (**e**,**f**) were determined by RT-qPCR and normalized to that of *OsUBQ5* (*LOC_Os01g22490*). Means and standard deviations were obtained from three biological replicates. Experiments were repeated three times with similar results. Red arrows indicate the average flowering date of HY.

We monitored the transcript levels of two rice florigen genes (*Hd3a* and *RFT1*) in developing leaves of HY and *rfs-2* plants harvested at ZT1 throughout the vegetative stages, until the HY plants flowered. *Hd3a* and *RFT1* transcripts gradually increased in HY with a peak at 8 WAS under SD and 15 WAS under LD conditions to trigger the transition to reproductive growth (Figure 3a–d) [34,35]. However, the expression of *Hd3a* and *RFT1* remained low in the *rfs-2* mutant throughout the experiment. The expression of *Ehd1* was similar to that of the florigen genes in HY and *rfs-2* plants (Figure 3e,f). These results suggest that mutation of *RFS* downregulates the expression of flowering genes, leading to late flowering in rice.

### 2.4. Expression Analysis of Flowering-Time Genes in the rfs Mutants

**Figure 4:**
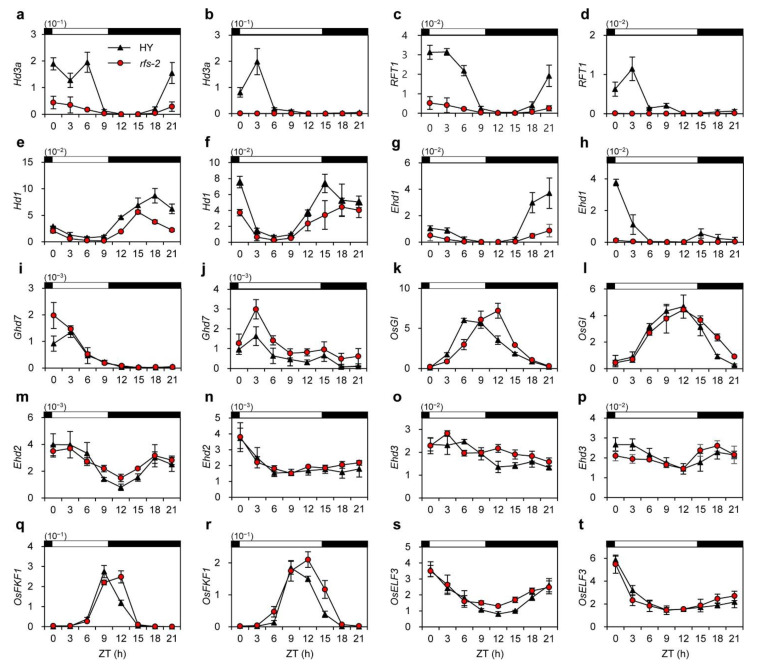
Diurnal expression of flowering genes in the *rfs-2* mutant. Rice plants were grown for 25 days after sowing (DAS) in SDs (**a**,**c**,**e**,**g**,**i**,**k**,**m**,**o**,**q**,**s**) and 80 DAS in LDs (**b**,**d**,**f**,**h**,**j**,**l**,**n**,**p**,**r**,**t**). Total RNA was isolated from the leaf blades collected every 3 h during a 24-h period. Transcript levels of *Hd3a* (**a**,**b**), *RFT1* (**c**,**d**), *Hd1* (**e**,**f**), *OsGI* (**g**,**h**), *Ehd1* (**i**,**j**), *Ghd7* (**k**,**l**), *Ehd2* (**m**,**n**), *Ehd3* (**o**,**p**), *OsFKF1* (**q**,**r**), and *OsELF3* (**s**,**t**) were determined by RT-qPCR and normalized to that of *OsUBQ5* (*LOC_Os01g22490*). The open and filled bars at the top of graphs represent light and dark periods, respectively. Means and standard deviations were obtained from three biological replicates. The experiments were repeated three times with similar results. ZT, zeitgeber time (hours after dawn).

To reveal the regulatory effect of *RFS* on flowering pathways in rice, we examined diurnal changes of the expression of flowering-time genes in the leaf blades of HY and *rfs-2* plants that were harvested during diurnal cycles, as shown in Figure 2b,c. The expression of *Hd3a* and *RFT1* was mostly suppressed in the *rfs-2* mutant, unlike the diurnal expression in HY under both SD and LD conditions (Figure 4a–d). Next, we measured the expression levels of *Ehd1* and *Hd1*, which encode upstream regulators of *Hd3a* and *RFT1* [3,4]. The expression level of *Hd1* in the *rfs-2* mutant was similar to that in HY under both photoperiods (Figure 4e,f). By contrast, *Ehd1* transcript levels were consistently lower in *rfs-2* leaves compared to HY at ZT21 and ZT0 in SD and LD conditions, respectively (Figure 4g,h). 

Previous genetic studies have revealed that diurnal expression of *Ehd1* is delicately regulated by various upstream regulators. We therefore examined the transcript levels of genes encoding upstream regulators of *Ehd1*: *OsGIGANTEA* (*OsGI*) [36], *Ghd7* [5], *Ehd2* [6,7,8], *Ehd3* [9], and rice *FLAVIN-BINDING, KELCH REPEAT, F-BOX 1* (*OsFKF1*) [37]. The expression of *Ghd7*, a negative regulator of *Ehd1*, was higher in the *rfs-2* mutant than in HY under LDs (Figure 4i,j). However, the transcript levels of positive regulators of *Ehd1*, including *OsGI*, *Ehd2*, *Ehd3*, and *OsFKF1*, were not altered in either photoperiod condition (Figure 4k–r). In addition, the expression of rice *EARLY FLOWERING 3* (*OsELF3*), a repressor of *Ghd7*, did not show any differences between HY and the *rfs-2* mutant (Figure 4s,t). These results suggest that RFS can repress the expression of *Ghd7* in LDs and activate the expression of *Ehd1* in both SD and LD conditions, thereby upregulating florigen genes in rice.

We further investigated whether *RFS* affects the expression of other epigenetic regulators including *SDG701* [13], *SDG708* [12], *OsTrx1* [14,15,16], *SDG724* [17], *SDG725* [18,19], and rice *VIN3-LIKE 2* (*OsVIL2*) [20,21]. These genes encode regulators that mediate histone methylation to promote rice flowering. The RT-qPCR analysis was performed on HY and *rfs-2* leaf blades sampled at ZT0 at 4 WAS and at ZT12 at 8 WAS in SD and LD conditions, respectively. There were no significant differences in the transcript levels of these genes between HY and *rfs-2* plants in either photoperiod condition (Figure S1). 

### 2.5. Histone Methylation Levels of Ehd1 and Ghd7 in the rfs Mutants 

A previous study reported that the *rfs* mutant showed global reductions of histone H3K4me3 and H3K27me3 by about 56% and 23%, respectively [31]. Therefore, we conducted a chromatin immunoprecipitation (ChIP) assay to determine whether RFS modifies the histone methylation level of flowering-time genes. The leaf blades of HY and *rfs-2* plants were collected at ZT1 at 9 WAS under LD conditions in the growth chamber. We speculated that downregulation of *Ehd1* in the *rfs-2* mutant might be caused by reduced levels of the activating mark H3K4me3 and upregulation of *Ghd7* in the *rfs-2* mutant might be caused by reduced levels of the repressive mark H3K27me3. 

**Figure 5:**
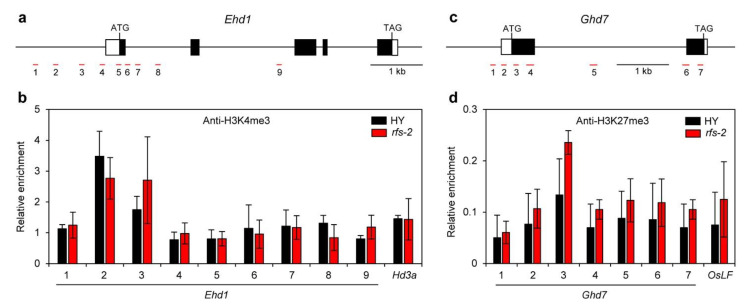
Histone modification patterns of differentially expressed genes in the *rfs-2* mutant. (**a**,**c**) Schematic representation of *Ehd1* (**a**) and *Ghd7* (**c**) loci. White and black boxes indicate untranslated regions and exons, respectively. Thick and thin black bars represent promoter and intron regions, respectively. Short red lines with numbers (1–9 for *Ehd1* and 1–7 for *Ghd7*) represent genomic DNA regions eluted from the protein–DNA complexes. The primers used for qPCR analysis of fragments are listed in Supplemental Table S1. Black scale bars = 1 kb. (**b**,**d**) Chromatin immunoprecipitation (ChIP) analysis of the H3K4me3 level on *Ehd1* (**b**) and the H3K27me3 level on *Ghd7* (**d**) in the 9-week-old HY and *rfs-2* plants grown in LD conditions at ZT1. *Hd3a* and *OsLF* were used as positive controls for the H3K4me3-enriched locus and the H3K27me3-enriched locus, respectively [18,25,27]. Means and standard deviations were obtained from three biological replicates. Experiments were repeated three times with similar results.

To test these hypotheses, we examined the enrichment of modified histone proteins on *Ehd1* and *Ghd7* chromatin using specific antibodies against H3K4me3 and H3K27me3. We used *Hd3a* and *OsLF* as positive controls for methylated histone-enriched loci. SDG701 deposits H3K4me3 on the *Hd3a* locus to activate *Hd3a* transcription [13]. The PRC2 complex targets the *OsLF* locus and deposits H3K27me3 to repress *OsLF* transcription [20,22]. We found that while H3K4me3 was highly enriched in the promoter region of *Ehd1* compared to the intergenic region, the H3K4me3 level in *Ehd1* did not differ between HY and the *rfs-2* mutant (Figure 5a,b). H3K27me3 in *Ghd7* was higher around the transcription start site than in the intergenic region. However, there was no significant difference in enrichment of H3K27me3 at the *Ghd7* locus between HY and the *rfs-2* mutant (Figure 5c,d). 

### 2.6. Agronomic Traits of the rfs Mutants

**Figure 6:**
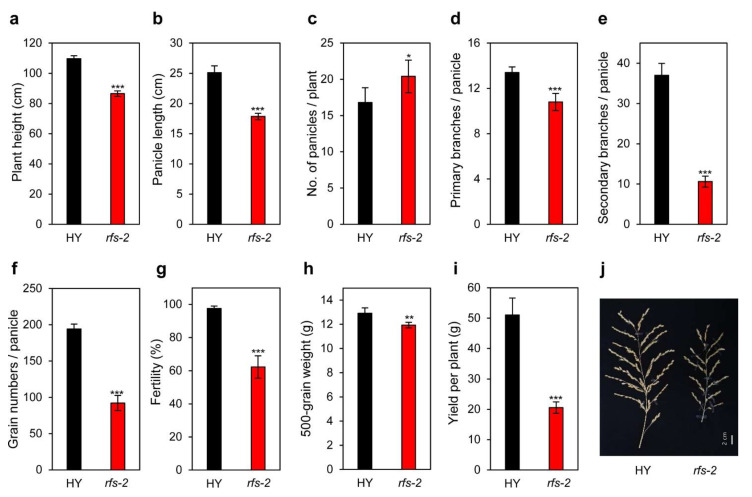
Agronomic traits assessed in the *rfs-2* mutant. Nine agronomic traits were examined and compared in the HY and *rfs-2* plants after harvest in the autumn. Plant height (**a**), panicle length (**b**), number of panicles per plant (**c**), number of primary branches per panicle (**d**), number of secondary branches per panicle (**e**), number of grains per panicle (**f**), spikelet fertility (**g**), 500-grain weight (**h**), yield per plant (**i**), and phenotype of panicles (**j**). Values are shown as means (*n* = 5) and error bars indicate standard deviation. Differences between means were compared using two-tailed Student’s *t*-tests (* *p* < 0.05, ** *p* < 0.01, *** *p* < 0.001).

To identify whether the *rfs* mutation affects grain yield and yield components, we evaluated agronomic traits, including plant height, number of panicles per plant, length of panicles, number of panicle branches per panicle, number of grains per panicle, spikelet fertility, 500-grain weight, and yield per plant, in HY and *rfs-2* plants grown in the paddy field under NLD conditions [38]. Plant height and panicle length of the *rfs-2* mutant were shorter than those of HY (Figure 6a,b). The *rfs-2* mutant had more panicles per plant compared to HY, but had fewer primary and secondary branches per panicle (Figure 6c–e,j). In addition, the number of grains per panicle, spikelet fertility, and 500-grain weight were significantly lower in the *rfs-2* mutant compared to HY (Figure 6f–h). The reduction of spikelet fertility in the *rfs-2* mutant might be due to delayed flowering, which results in later grain filling, when conditions tend to be unfavorable due to lower temperatures. Consequently, total grain yield was lower in the *rfs-2* mutant due to the reduction of yield components, including spikelet fertility, grain number, and 500-grain weight, although the mutant plants had more panicles per plant compared with HY (Figure 6i). 

## 3. Discussion

### 3.1. Regulatory Roles of RFS in Flowering-Time Pathways

Studies of various mutant alleles of *RFS* have revealed that *RFS* functions in multiple aspects of plant development, including crown root development, seedling development, and leaf morphogenesis [28,29,30]. In this study, we found that *RFS* also affects rice flowering time. Mutation of *RFS* delayed flowering under SD and LD conditions (Figure 1). Moreover, expression of two rice florigen genes, *Hd3a* and *RFT1*, maintained at low levels in *rfs-2* mutant whereas their transcript levels were elevated in HY throughout plant growth (Figure 3). In HY, *RFS* expression showed a diurnal rhythm with a peak at ZT15 and ZT0 under SD, and LD conditions, respectively (Figure 2b,c). The expression of *Ehd1* subsequently peaked at ZT18 in SDs and at ZT0 in LDs (Figure 4g,h). Finally, *Hd3a* and *RFT1* transcript levels were strongly upregulated at ZT21 and ZT3 under SD and LD conditions, respectively (Figure 4a–d). Our findings strongly suggest that *RFS* is a floral inducer in rice.

**Figure 7:**
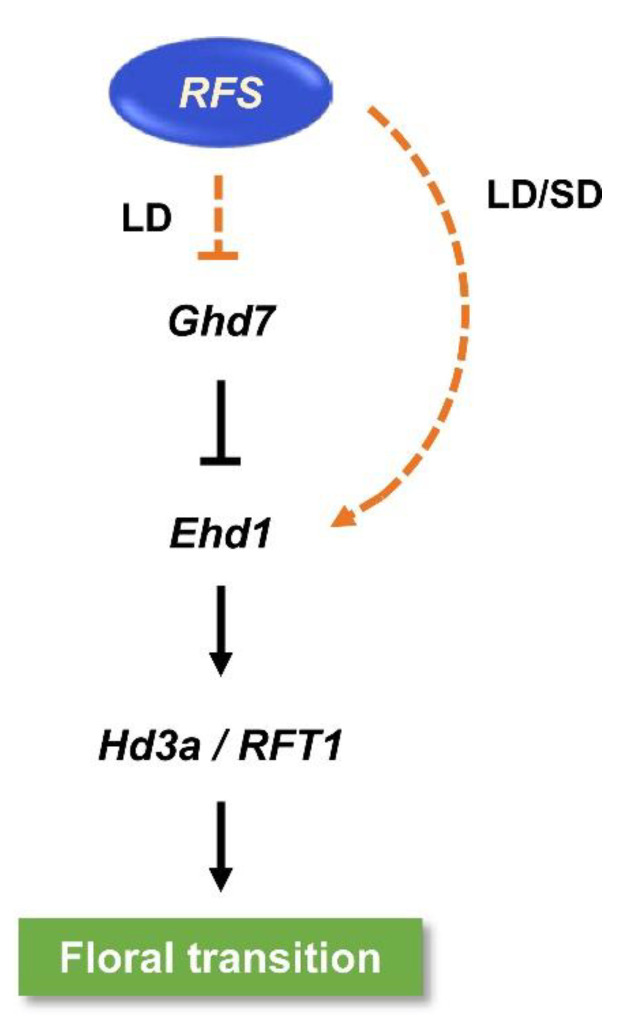
Proposed model of the flowering regulatory pathway controlled by *RFS*. *RFS* downregulates *Ghd7* in LDs and upregulates *Ehd1* in LD and SD conditions to induce the floral transition. Arrows represent upregulation, and lines ending with bars represent downregulation. Solid and dashed lines indicate direct and indirect regulation, respectively.

Mutation of *RFS* decreased the expression of the florigen genes *Hd3a* and *RFT1*, which are rice orthologs of *Arabidopsis FT*, under SD and LD conditions (Figure 4a–d). In *Arabidopsis*, the CHD3 protein PKL promotes flowering through activation of the *FT* locus, suggesting that CHD3 chromatin-remodeling factors have conserved functions in flowering plants. However, among the upstream regulators of *Hd3a* and *RFT1*, there were no differences in *OsGI* and *Hd1* expression between HY and *rfs-2* plants (Figure 4e,f,k,l), but the transcript levels of *Ghd7* and *Ehd1* in the *rfs-2* mutant differed from those in HY (Figure 4g–j). These observations implied that *RFS* does not participate in the *OsGI–Hd1–Hd3a* regulatory pathway, which is similar to the *Arabidopsis GI–CO–FT* module. Instead, *RFS* may regulate flowering time through rice-specific flowering pathways involving *Ghd7* and *Ehd1* (Figure 7). Although CHD3 chromatin remodeling factors are involved in the regulation of flowering in rice and *Arabidopsis*, the regulatory pathways might differ between the two species.

### 3.2. RFS May Control Expression of Flowering-Time Genes via Histone Modification

In *Arabidopsis*, PKL mediates deposition of H3K27me3 to repress target genes [39,40,41,42], and mutation of *CHR4* changes H3K27me3 and H3K4me3 levels [27]. Moreover, RFS is responsible for modification of H3K27me3 and H3K4me3 to control gene expression [31,32,43]. We therefore speculated that RFS controls the expression of *Ghd7* and *Ehd1* through H3K27me3, and H3K4me3, respectively. However, our ChIP analysis showed no apparent differences in H3K4me3 levels of *Ehd1* or H3K27me3 levels of *Ghd7* between HY and the *rfs-2* mutant (Figure 5). One possible explanation for this is that RFS regulates *Ehd1* and *Ghd7* via other types of modifications, such as histone acetylation. In mammalian cells, CHD3 is a component of the nucleosome remodeling and deacetylase (NURD) complex, which is associated with histone deacetylation, resulting in transcriptional repression [44,45]. In addition, RFS might control an unidentified upstream regulator of *Ehd1* and *Ghd7*. A previous study proposed that OsTrx1 may be an upstream regulator of *Ghd7* [14]. In the absence of OsTrx1, which activates target genes by adding the H3K4me3 mark, the expression of *Ghd7* increased, suggesting that *Ghd7* expression might be indirectly controlled by OsTrx1. Therefore, further analysis and exploration will be required to elucidate the involvement of RFS in flowering.

ATP-dependent chromatin remodeling factors act as multimeric complexes in various organisms. In this study, we could not observe significant alteration in transcript levels of other epigenetic regulators that promote rice flowering, including *SDG701* [13], *SDG708* [12], *OsTrx1* [14,15,16], *SDG724* [17], *SDG725* [18,19], and *OsVIL2* [20,21] between HY and *rfs-2* (Figure S1). These results suggest that *RFS* is not directly involved in the transcriptional regulation of these epigenetic regulators. Therefore, we could not exclude the possibility that RFS recruits these regulators to modify the histone methylation of flowering-time genes. Recent biochemical approaches using immunoprecipitation and mass spectrometry have identified the components of ISWI and SWI/SNF chromatin remodeling complexes in plants [46,47]. In *Arabidopsis*, PKL and CHR4 recruit several transcription factors and cofactors for proper epigenetic regulation during development [25,26,27,48]. In rice, the components of complexes that associate with CHD3 have not yet been elucidated. Therefore, it will be interesting to find the components that physically interact with RFS during rice development, including flowering, crown root development, seedling development, and leaf morphogenesis.

### 3.3. RFS Is Involved in Inflorescence Development

CHR4 plays diverse roles in the inflorescence meristem to promote flowering in *Arabidopsis* [27]. *CHR4*-deficient plants show an accelerated transition from the vegetative phase to bolting, but a delay in the formation of floral primordia. CHR4 interacts with MADS, SQUAMOSA promoter-binding protein-like (SPL), and APETALA2 (AP2)-type transcription factors, which regulate the floral transition and floral meristem identity [24,49]. In addition, RNA-sequencing and ChIP sequencing have revealed that CHR4 mediates the response to endogenous flowering pathways in the inflorescence meristem by controlling the expression of floral regulators [27]. In rice, overexpression of *ABERRANT PANICLE ORGANIZATION 1* (*APO1*), which encodes an F-box protein, causes a precocious transition of the inflorescence meristem to the spikelet meristem, reducing the number of panicle branches [50]. The *rfs-2* mutant had significantly decreased grain yield compared to HY (Figure 6i). Based on these findings, we speculated that RFS might control other developmental processes, as well as flowering. Among the agronomic traits that affect grain yield, the number of secondary panicle branches was markedly reduced in the *rfs-2* mutant (Figure 6e), implicating *RFS* in inflorescence meristem fate. Because the inflorescence architecture of rice is closely related to yield, the role of RFS in the transition from the vegetative meristem to the inflorescence meristem will be an interesting topic for future studies.

## 4. Materials and Methods

### 4.1. Plant Materials and Growth Conditions

The *rfs-1* and *rfs-2* mutants used in this study were reported previously [32]. Rice plants were grown in the paddy field under NLD conditions (≥14 h sunlight/day, 37° N latitude) in Suwon, Republic of Korea. To perform experiments under controlled day-length conditions, rice plants were grown in growth chambers under LD (14.5-h light and 9.5-h dark) or SD (10-h light and 14-h dark) conditions at 30 °C in the light and 24 °C in the dark.

### 4.2. RNA Extraction and Reverse Transcription-Quantitative PCR (RT-qPCR) Analysis

Total RNA was isolated using an RNA extraction kit (MG Med, Seoul, Korea) according to the manufacturer’s manual. After RNA quantification, cDNA was synthesized from 2 µg of total RNA by M-MLV reverse transcriptase (Promega, Madison, WI, USA) at 42 °C for 1 h after priming with oligo(dT) (Promega, Madison, WI, USA) at 70 °C for 5 min. Prepared cDNAs were diluted to 100 µL with distilled water and then used as templates for RT-qPCR. RT-qPCR was performed with a LightCycler 480 (Roche, Basel, Switzerland) using 2× GoTaq master mix (Promega, Madison, WI, USA) in a 20-µL reaction volume. Rice *UBIQUITIN 5* (*LOC_Os01g22490*) served as an internal control for relative quantification. The primer sequences used for RT-qPCR are listed in Supplemental Table S1.

### 4.3. Chromatin Immunoprecipitation (ChIP) Assay

ChIP was performed as previously described [51]. Leaf blades of 63-d-old plants grown in the LD growth chamber were harvested at ZT1. One gram of leaves was crosslinked in 1% formaldehyde under vacuum. After isolation of nuclei, chromatin was sheared into 500–1000 bp in length by sonication and then immunoprecipitated with anti-H3K4me3 (Millipore, Temecula, CA, USA) or anti-H3K27me3 (Millipore, Temecula, CA, USA) antibodies. The immunoprecipitated products and 5% of input chromatin were reverse-crosslinked at 65 °C and eluted with QIAquick PCR purification kit (Qiagen, Hilden, Germany). Finally, the precipitated DNA was quantified by qPCR with the primers listed in Supplemental Table S1.

### 4.4. Measurement of Agronomic Traits

To investigate agronomic traits, HY and *rfs-2* plants were grown in the paddy field under NLD conditions. Plant height was measured just after heading. The other traits, such as panicle length, the number of panicle branches, the number of grains, fertility, 500-grain weight, and yield per plant, were examined after harvest. The panicles of main tillers were used to analyze panicle length, the number of panicle branches, and the number of grains.

## 5. Conclusions

In this study, we show that a CHD3/Mi-2 chromatin remodeling factor, RFS, is involved in the regulation of flowering induction in rice. *RFS* loss-of-function mutants exhibited a late-flowering phenotype under SD and LD conditions. *RFS* promotes the expression of two rice florigen genes, *Hd3a* and *RFT1*, through downregulation of *Ghd7* and upregulation of *Ehd1*, thereby promoting floral induction. This study, thus, provides evidence that a chromatin remodeling factor plays crucial roles in rice flowering.

## Data Availability

The data presented in this study are available in the article.

## Supplementary Materials

Supplementary materials can be found at https://www.mdpi.com/1422-0067/22/3/1303/s1, Figure S1. Expression level of epigenetic regulators of rice flowering in the *rfs-2* mutant; Table S1. List of primers used in this study.

## Abbreviations

RFS	Rolled Fine Striped
SD	Short day
LD	Long day
NLD	Natural long day
H3K4me3	Trimethylation of histone H3 lysine 4
H3K27me3	Trimethylation of histone H3 lysine 27
SL	Seolak
HY	Hwayoung
ZT	Zeitgeber time
DAS	Days after sowing
RT-qPCR	Reverse transcription quantitative PCR
ChIP	Chromatin Immunoprecipitation
