# Supplementary material for: The Rice CHD3/Mi-2 Chromatin Remodeling Factor Rolled Fine Striped Promotes Flowering Independent of Photoperiod
Source: Int J Mol Sci. 2021 Jan 28;22(3):1303. doi: 10.3390/ijms22031303 (PMC7865970; doi:10.3390/ijms22031303)
Supplement: Supplementary file 1 [file ijms-22-01303-s001.pdf]

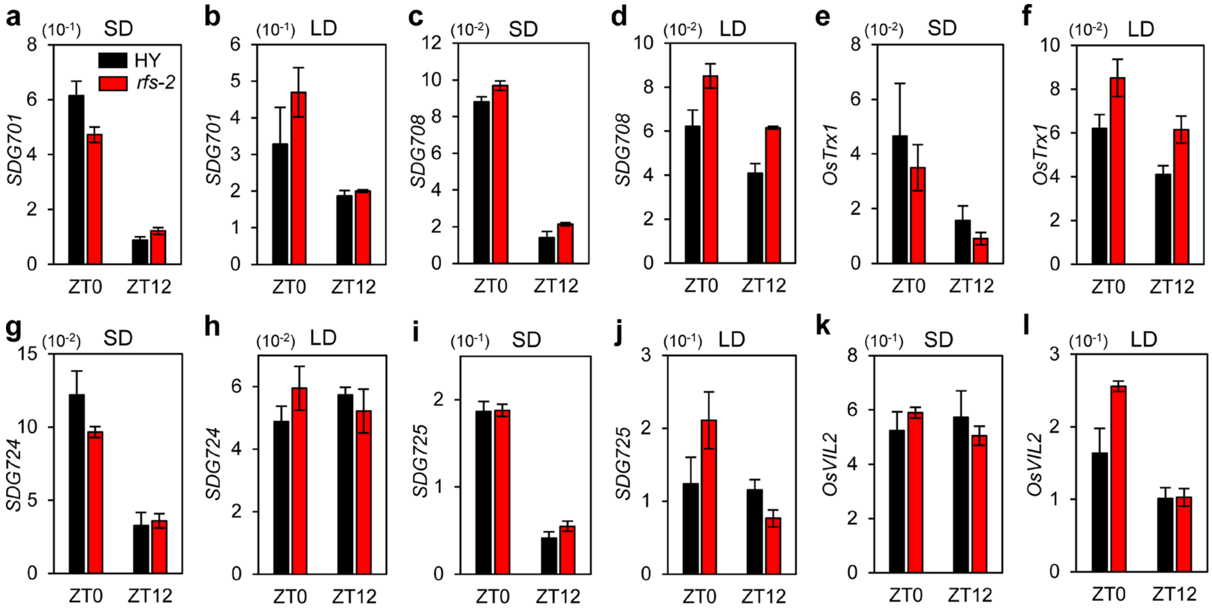


**Figure S1**. Expression level of epigenetic regulators of rice flowering in the *rfs-2* mutant.
Total RNA was isolated at ZT0 and ZT12 from the leaf blades of HY and *rfs-2* plants grown in the growth chambers for 4 weeks and 8 weeks in SD (**a**,**c**,**e**,**g**,**i**,**k**) and LD (**b**,**d**,**f**,**h**,**j**,**l**) conditions, respectively. Transcript levels of *SDG701* (**a**,**b**), *SDG708* (**c**,**d**), *OsTrx1* (**e**,**f**), *SDG724* (**g**,**h**), *SDG725* (**l**,**j**), and *OsVIL2* (**k**,**l**) were determined by RT-qPCR and normalized to that of *OsUBQ5* (*LOC_Os01g22490*). Means and standard deviations were obtained from three biological replicates. Experiments were repeated three times with similar results. ZT, zeitgeber time (hours after dawn). HY, Hwayoung cultivar, which is the parental line of the *rfs-2* mutant.

**Table S1.** List of primers used in this study.

| | Primer name | Forward primers (5'→3') | Reverse primers (5'→3') |
|---|---|---|---|
| RT-qPCR | *Ubiquitin 5* | ACCACTTCGACCGCCACTACT | ACGCCTAAGCCTGCTGGTT |
| | *RFS* | CGTCTCTCTCTCCCCAAGGA | CAGGGAGGAAGCTTGCTGAA |
| | *Hd3a* | CTTCAACACCAAGGACTTCGC | TAGTGAGCATGCAGCAGATCG |
| | *RFT1* | TGACCTAGATTCAAAGTCTAATCCTT | TGCCGGCCATGTCAAATTAATAAC |
| | *Ehd1* | GTTGCCAGTCATCTGCAGAA | GGATGTGGATCATGAGACAT |
| | *Hd1* | TCAGCAACAGCATATCTTTCTCATCA | TCTGGAATTTGGCATATCTATCACC |
| | *OsGI* | ATCGTTCTGCAGGCCGAGA | TCACCAATGCTTCTGGGCTAT |
| | *Ghd7* | AGGTGCTACGAGAAGCAAATCC | GGGCCTCATCTCGGCATAG |
| | *Ehd2* | CGACGACAATAGCTCGATCGC | GTGCATGGTCACGGAGCCTT |
| | *Ehd3* | GGACCACCTCGTCACCTACAA | CGCCGTTGGCCATGAG |
| | *OsFKF1* | ATGGCACAGTTCATGTACCCTGGA | TCCTTGGTGAGGTCAAGCAGGAAT |
| | *OsELF3* | ACCACTTCGACCGCCACTACT | ACGCCTAAGCCTGCTGGTT |
| | *SDG701* | CACAAGAGCGAGTCTATGGC | GCATCCATCATTGGAGTACC |
| | *SDG708* | GAATGTTGTGGGTATCTGTG | CTTATCTCGTCGTACAGGCT |
| | *OsTrx1* | GGTCACATCAGAAGATGGAA | CACCATAGCATCTAGCATGTA |
| | *SDG724* | CTGTGGACTTTGATCTGCCG | GGATGATCCAGAAGGGGTAC |
| | *SDG725* | CCTATTGACAGTGAGCACAT | AGCATTGTCACCTTCCGCTG |
| | *OsVIL2* | GATGCGTGGCTGAAGTCAAAC | GCAGCTTCTTTCAGTACCAC |
| ChIP | *Ehd1*_P1 | GACCGAACCCGACCCGTTT | GTGTATGCGATTGCGCGCTT |
| | *Ehd1*_P2 | GCCGTCCACAACCTGAGTTA | GCTAGCTAGGTAAGGAAGAA |
| | *Ehd1*_P3 | CGTCATGATCATATCAACGG | GTAAATACTATATACGACAGG |
| | *Ehd1*_P4 | CTGTGTCTAGCTTGCACTAC | TGCATGATGCATGGAATGCG |
| | *Ehd1*_P5 | CACCGAGAGCTGTGGCCTTA | AGAAGTAAATCTTCCATGACTGACA |
| | *Ehd1*_P6 | TCATCGATGACGACTGTTCA | TATATAATCTTAATTCGCATC |
| | *Ehd1*_P7 | ACTACACACGTGTCCATGCA | ACTGACTGAACTTAAATAGTAG |
| | *Ehd1*_P8 | AAAGATGATCAGCTCTGTGG | TACCCTCCAAGACTTCGATT |
| | *Ehd1*_P9 | AACCCCATCTACAACTGGCT | AACACTTCACTAGAGCAGCCC |
| | *Ghd7*_P1 | ATGGGTAGTGAAGTCCAGCC | TCTGTGGATGGATTTGGTCC |
| | *Ghd7*_P2 | ATCCCCAACTTGCCCTGTCT | TAGCTATAGCAGGTGAGGTC |
| | *Ghd7*_P3 | TCATGTCGATGGGACCAGCA | GGAAGACGAAGGGGAATCCA |
| | *Ghd7*_P4 | GCCTGGCTGTTCGATGACTA | AGGGAGACGTCGAACGTGA |
| | *Ghd7*_P5 | CATACGGATCCAGCCTCTGT | ACTCGAAAAGCACACGCGAC |
| | *Ghd7*_P6 | CGATGATTAATTGTATTCGAGC | TCAAGCTCTCCCCATCATCG |
| | *Ghd7*_P7 | TGATCAGGAAGCTGTCGCAC | TAGTCAGTGGTATATACGCAC |
| | *Hd3a* | TTGTGGTTGGTAGGGTTG | AAGGGTGTAGAATGTCCTCATG |
| | *OsLF* | CAGCATTTTTGGTTGGGAGT | ACCCGAAGGCGTCCATGT |
